# Development, Maintenance, and Reversal of Multiple Drug Resistance: At the Crossroads of TFPI1, ABC Transporters, and HIF1α

**DOI:** 10.3390/cancers7040877

**Published:** 2015-10-16

**Authors:** Terra Arnason, Troy Harkness

**Affiliations:** 1 Department of Medicine, University of Saskatchewan, Saskatoon, SK S7N 0W8, Canada; 2 Department of Anatomy and Cell Biology, University of Saskatchewan, Saskatoon, SK S7N 0W8, Canada; troy.harkness@usask.ca

**Keywords:** multiple drug resistance/MDR, TFPI, hypoxia, HIF1, breast cancer

## Abstract

Early detection and improved therapies for many cancers are enhancing survival rates. Although many cytotoxic therapies are approved for aggressive or metastatic cancer; response rates are low and acquisition of *de novo* resistance is virtually universal. For decades; chemotherapeutic treatments for cancer have included anthracyclines such as Doxorubicin (DOX); and its use in aggressive tumors appears to remain a viable option; but drug resistance arises against DOX; as for all other classes of compounds. Our recent work suggests the anticoagulant protein Tissue Factor Pathway Inhibitor 1α (TFPI1α) plays a role in driving the development of multiple drug resistance (MDR); but not maintenance; of the MDR state. Other factors; such as the ABC transporter drug efflux pumps MDR-1/P-gp (ABCB1) and BCRP (ABCG2); are required for MDR maintenance; as well as development. The patient population struggling with therapeutic resistance specifically requires novel treatment options to resensitize these tumor cells to therapy. In this review we discuss the development, maintenance, and reversal of MDR as three distinct phases of cancer biology. Possible means to exploit these stages to reverse MDR will be explored. Early molecular detection of MDR cancers before clinical failure has the potential to offer new approaches to fighting MDR cancer.

## 1. Introduction

Of the many scientific advances made in our lifetime, effective cancer treatment and control still remains elusive. Considering breast cancer, approximately one in eight women will face this disease sometime during their life [1] with most recent estimates showing that 23,800 Canadian women developed, and 5000 died from breast cancer in 2013 [2]. Malignancies of the breast are an example of a common cancer that frequently returns years after initial therapy, but in a treatment-resistant form, despite the combined use of aggressive adjuvant and neoadjuvant approaches [3,4,5,6]. These tumors then display a confounding multiple drug resistance (MDR) [7]. Some mechanisms regulating MDR are known, and frequently include increased expression of drug efflux transporters [8], yet inhibitors against these processes have not yielded clinical benefits [9]. Given the recurrence rate of treatment-resistant breast tumors and the limited number of therapies available, there is clearly both need and urgency to find novel strategies, early detection, and efficient reversal of MDR cancers.

## 2. Detection of Treatment Resistance in Breast Cancer Therapy

Currently, detection of MDR cancers remains a clinical diagnosis, however, there is great interest in validating biomarkers that can provide clinical direction by identifying those individuals at risk of having or developing treatment resistance (reviewed in [10,11]). Ideally, the appearance of such proteins would provide early and accurate biochemical detection of recrudescent MDR disease before its resistant nature is clinically apparent, while their loss will provide evidence of tumor resensitization to drug therapy. There are many candidate biomarkers identified in the literature, but none are yet being routinely used for clinical decisions. It remains unclear if the growing list of activated or repressed proteins and genes linked to MDR [12,13,14] are causing, or responding, to MDR. General mechanisms of MDR often involve increased expression of ATP-binding efflux pumps that lower effective intracellular chemotherapeutic drug concentrations (include Multiple Drug Resistant protein-1 (MDR-1) and Breast Cancer Resistance Protein (BCRP)), and this applies to any cancer, whether the resistance is inherent or acquired [15,16,17]. Although there are 49 family members of the ABC transporters divided into 8 distinct groups [18], their importance in drug resistance is not equal. Three subfamilies have been identified as relevant for MDR: MDR-1 (ABCB1) and BCRP (ABCG2) predominantly pump large hydrophobic, positively charged amphiphilic compounds including major chemotherapeutic agents used in breast cancer (e.g., DOX, mitoxantrone, or verapamil) out of the cell, while the multidrug resistance proteins (MRPs) found in the ABCC subfamily, drive out hydrophobic uncharged molecules, as well as water-soluble anionic compounds [19]. Use of DOX to treat a wide variety of tumors has revealed multiple resistance mechanisms, including over-expression of proteins mediating hypoxia (HIF1α; a hypoxic microtumoural environment is potentially present in ~40% of breast tumours [20,21]), invasion (Twist1), and transcriptional activation of survival pathways (HIF1α, NF-κB, MAPK), or reductions in checkpoint, DNA repair or cell cycle arrest proteins (p53, p21) [8]. The cellular mechanisms recruited for treatment resistance are clearly complex and multifactorial. To appreciate the nuances of this event, we consider MDR as three biological stages: development, maintenance, and reversal.

## 3. Development of MDR

**Figure 1:**
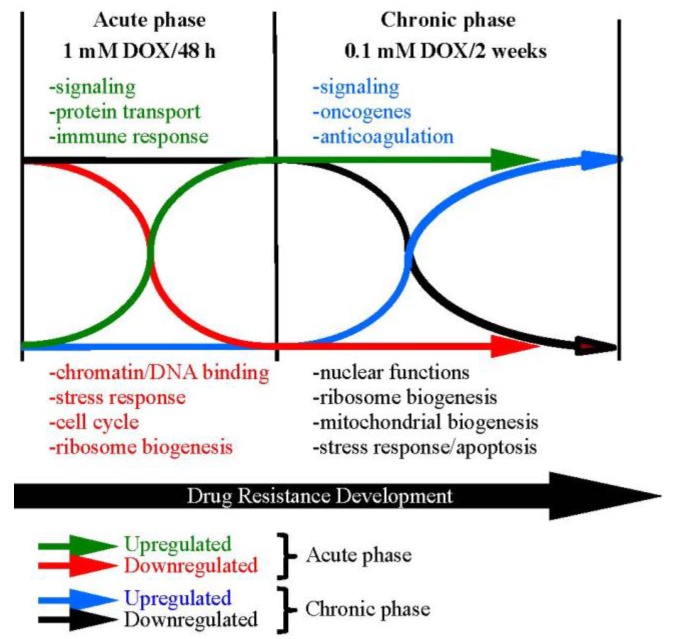
Microarray expression changes grouped by biological function detected during the acute and chronic selection phases for Doxorubicin resistance in MCF7 breast cancer cells.

To begin to understand how cancer cells develop drug resistance, we measured transcriptional changes as cells respond to an acute 48 h exposure to 1 μM DOX, followed by a two week chronic exposure to 100 nM DOX in MCF7 breast cancer cell lines to select for DOX resistant (DOX^Res^) cells [22]. Our previous studies demonstrated that DOX^Res^ cells developed in this manner have an extended repertoire of drug-resistant phenotypes, including resistance to Troglitazone [22,23,24] and Metformin (unpublished), supporting the MDR-label. Cells were harvested prior to treatment and following the acute and chronic exposure to DOX. We observed that 464 genes (396 up-regulated and 68 down-regulated) were differentially expressed following acute exposure to DOX, but returned to baseline levels following the two-week chronic selection period. These genes reflected those that transiently respond to DOX, and are not necessarily required for the development of MDR. However, we observed genes that remained differentially expressed (154 up, 118 down) or became differentially expressed (53 up, 102 down) following chronic exposure [22]. Gene networks expected to be involved with MDR development were identified, such as increased signaling, protein transport, and oncogene expression, as well as decreased stress response, chromatin dynamics, cell cycle control, and ribosome and mitochondrial dynamics (Figure 1).

### 3.1. Tissue Factor Pathway Inhibitors and Tumor Suppression

We were particularly interested in the novel up-regulation of a suite of genes involved in anticoagulation during the chronic selection period. The anticoagulant protein Tissue Factor Pathway Inhibitor 1 (TFPI1, or simply TFPI) was among the most highly up-regulated genes induced during chronic DOX exposure [22]. The evolutionarily-conserved TFPI family of proteins are Kunitz-type serine proteases that prevent the cleavage of prothrombin by a pathway controlled by Tissue Factor (TF) [25]. TFPI1 is a complex protein that is expressed as three potential isoforms, TFPI1α, β, and γ [26]. TFPI1α and β are the predominant forms with the α isoform expressed approximately 10-fold more than the β isoform [27], while expression of the γ isoform is in question [28]. TFPI1α and β are both expressed by endothelial cells, but only TFPIα is expressed in platelets [26]. Furthermore, TFPI1α is believed to be primarily a secreted protein, with some plasma membrane cell surface binding [29], while TFPI1β encodes a distinct C-terminus and remains exclusively associated with cell surfaces via a glycosylphosphatidylinisotol (GPI)-attachment motif.

The coagulation pathway, activated by TF, culminates in the production of thrombin, which activates platelets by cleaving fibrinogen to fibrin leading to clot formation [30]. However, increased TF and thrombin expression has also been found to correlate with increased angiogenesis, metastasis and tumor invasion [31], likely through cleavage of protease-activated receptors that facilitate the transcription of angiogenic factors. It was also observed that TF expression, specifically within the microtumoral environment, is a critical determinant of cancer progression [32]. In fact, hypercoagulation within the peripheral circulatory system is observed to occur in many malignancies [33,34] and individuals presenting with an unexplained blood clot are generally actively investigated for an occult malignancy. Treatment of cancer patients using anticoagulents, such as Heparin, can prevent thromboembolism [35,36], but the effects using anticoagulation drugs in cancer patients are short-lived and may cause evasive resistance by induction of secondary hypoxic pathways [37,38].

Although we found TFPI1 to be highly expressed in DOX^Res^ MCF7 cells, with additional evidence (discussed below) suggesting it may facilitate tumor progression, a contrasting body of evidence exists *in vitro* and *in vivo* to warrant consideration of TFPI1 as a tumor suppressor [39,40,41,42,43,44,45]. For example, *in vitro* TFPI1 silencing in breast cancer cells increased invasive tumor growth while TFPI1 overexpression enhanced apoptosis [41,43]. Consistent with this, an *in vivo* study of mice lacking both major TFPI1 isoforms (α and β) demonstrated that TFPI1 deficiency lead to increased metastasis, which was proposed to be due to increased TF-dependent thrombin synthesis [45]. A recent study analyzing microarray data of TFPI1 expression in human cancer patients showed a correlation between low TFPI1 levels and worse patient outcomes [46]. This evidence points towards TFPI1 as a tumor suppressor.

Similarly, the TFPI1 homolog, TFPI2, is also considered a tumor suppressor [47] due to several lines of evidence: its promoter has been shown repeatedly to be hypermethylated and silenced in cancer cell lines compared to normal cells, it is silenced in multiple cancer cell lines and patient samples, and its *in vitro* knockdown resulted in cancer cell migration and invasion [48,49,50,51,52,53]. A recent paper revealed that TFPI2 (also known as PP5) [54], which is also known to have protein phosphatase activity, binds to the ABC multidrug transporter MDR-1/ABCB1 to dephosphorylate and reduce the transporter’s activity; PP5/TFPI2 silencing resulted in increased MDR-1 expression and function [55]. This suggests that TFPI2 could be important for blocking drug resistance. Considering its tight correlation with tumor progression, TFPI2 has thus been labeled a DNA-methylation biomarker [56].

### 3.2. Tissue Factor Pathway Inhibitor Protein and Tumor Progression

There are several lines of independent evidence that support a role for TFPI1 in tumor progression that are as compelling as the evidence showing its contribution to cancer suppression. While overexpression of two of the TFPI1 isoforms, TFPIα and β, in breast cancer cells resulted in increased apoptosis of tumor cells [41,43], a recent microarray analysis of breast cancer cells overexpressing TFPIα or β *in vitro* resulted in differential expression of many genes involved in cell development, such as cell growth, adhesion, migration, invasion, and apoptosis [42]. The authors concluded that the expression profile observed in TFPI1β expressing cells was consistent with relapse-free survival, suggesting that TFPI1β could have therapeutic potential. Differentially-expressed genes diverged between the two isoforms, with those upregulated by TFPI1β involved in processes such as carbohydrate metabolism, free radical scavenging, lipid metabolism, and cellular response to therapeutics. In contrast, the top upregulated genes after TFPI1α expression predominantly included those involved in immune response, which have been shown to be involved in cancer progression [57]. Interestingly, although it was concluded from these analyses that TFPI1β may be required for tumor suppression, both TFPI1α and β induced the activity of cancer-driving transcription factors Elk-1, NF-κB, and the phosphorylation of AKT, highly suggestive of a role in cancer progression.

The idea that TFPI1 may be involved with tumor progression (rather than acting as a tumor suppressor) is further supported by the observations that TFPI1 mRNA and protein expression is found increased *in vivo*, in tumors isolated from patients with more aggressive cancers [46,58,59]. TFPI1 has also been observed highly upregulated in cell lines derived from aggressive tumors [60,61,62,63]. The apparent paradox of having TFPI1 act as a tumor suppressor, yet be highly expressed in aggressive tumors was recently noted, and it was suggested that this could be explained if increased TFPI1 in more aggressive tumors provides a means to reduce the increased TF and thrombin activity in these cells, thus acting as a marker of aggressive tumors [45,46]. While possible, other explanations also exist and are considered below.

### 3.3. Elevated TFPI1 is Associated with Multiple Drug Resistance Development in Vitro and with MDR in Patient Samples

The controversial role of TFPI1 in tumor progression, together with our finding that only TFPI1α, but neither TFPI1β nor TFPI2, was specifically up-regulated in MCF7 DOX^Res^ cells, prompted us to focus on TFPI1α. We asked whether TFPI1 overexpression is an attempt by the cell to block the tumorigenic potential of increased thrombin levels, or whether the resultant inhibition of the angiogenic pathway leads to a hypoxic state and the subsequent expression of HIF1α, a potent driver of angiogenesis and invasive cancer. Our observations that TFPI1 abundance and subcellular translocation corresponded with MDR development in breast cancer cells supported its role as a tumor promoter. In our analysis of elevated TFPI1 expression in MCF7 DOX^Res^ cells [22], we first observed that thrombin protein levels were decreased in parallel with upregulated TFPI1 mRNA and protein, suggesting the increased TFPI1 protein was indeed active and functioning normally in the anticoagulant pathway. Using fluorescent microscopy we observed weak TFPI1 expression in parental cells, and increased perinuclear and striking nucleolar accumulation in selected cells. Considering that TFPI1α is thought to be localized to the cell surface and to be excreted into circulation [26,29], relocalization of TFPI1 from the cell surface to the nucleolus provides an explanation for why elevated TFPI1 levels alter the transcription of so many genes [42]. We are currently investigating this observation further, but find it important to note that the highly basic C-terminal 9 amino acid sequence found only in TFPI1α contains a perfect monopartite nuclear localization signal.

TFPI1 overexpression is not specific to the MCF7 cell lines that have undergone selection for resistance to DOX, as we have demonstrated that TFPI1 protein levels were elevated in all MDR cells tested, including human K562 myelogenous leukemia cells and human colon adenocarcinoma Colo201 cells we selected for DOX resistance, and rat glioblastoma treatment sensitive and resistant C6 and F98 cells, respectively [64]. TFPI1 protein induction began after 24 h of 1 μM DOX exposure, at the same rate as the ABC transporter BCRP, and was dependent on DOX concentration, as TFPI1 was mildly overexpressed after 96 h of 100 nM DOX exposure. This suggests that TFPI1 is an early player in development of resistance to DOX. TFPI1 was not required for MDR maintenance since silencing of TFPI1 in MCF7 DOX^Res^ cells did not sensitize the cells to DOX re-exposure [22].

To gather a greater understanding of TFPI1’s role in cancer progression, we overexpressed the TFPI1α isoform in MCF7 parental cells. We predicted that if TFPI1 is blocking the induction of the angiogenic pathway mediated through thrombin, then it is likely the generation of a TFPI1-dependent hypoxic-like state, even under normoxic *in vitro* conditions, and the subsequent induction of protein like HIF1α, that is driving aggressive tumor growth [65,66,67]. This is indeed what we observed, as well as overexpression of additional canonical cancer driving proteins, such as PAR-1, c-MYC, c-SRC, HDAC2, and increased post-translational modifications of histone H3 (H3K14^Ac^ and H3K79^me2^). The induction of HIF1α is apparently linked to drug resistance, having shown that cells surviving hypoxia (1% O_2_ for 48 h) expressed markers of MDR, such as BCRP, MDR-1, c-MYC, and AKT phosphorylation, and were completely resistant to DOX [22]. Moreover, overexpression of TFPI1α in MCF7 parental cells for 24 h increased the resistance of these cells to 1 μM DOX. Lastly, we validated our observations using patient samples obtained from Agilent expression arrays. We sorted 1223 datasets from breast (529), ovarian (539), or colon (155) tumors according to expression of BCRP. Those datasets with high BCRP expression also had elevated MDR-1, HIF1α, and TFPI1 expression. In contrast, and as expected for a role in tumor suppression, TFPI2 expression was decreased. Our data is the first to show that increased TFPI1 in aggressive tumors is likely a response to chemotherapy exposure, and one that plays an early role in facilitating MDR development.

## 4. Maintenance of MDR

### 4.1. The Role of ABC Efflux Pumps in MDR Maintenance

Alluding to our concept of three distinct phases of MDR, maintenance (*in vitro*) refers to sustained treatment resistance without ongoing selection pressure (*i.e.*, stable MCF7 DOX^Res^ phenotype without ongoing DOX exposure). As noted above, a common theme allowing for resistance to chemotherapy incorporates an increased efflux of drug from the cell. This was first observed in 1973 [68], and the discovery that MDR-1/P-gp/ABCB1 was over-expressed in those cells that were resistant to therapy in 1976 [69] appeared to solidify this theory within the field (reviewed in [70]). Silencing of MDR-1 in chemoresistant cell lines *in vitro* supported this as it was consistently shown to increase sensitivity to DOX to varying degrees [23,71,72]. Other drug efflux pumps, such as BCRP/ABCG2, are also thought to play pivotal roles in MDR, and silencing of BCRP in cells overexpressing BCRP also consistently sensitized cells to subsequent exposure to chemotherapeutic drugs [73,74,75,76]. Surprisingly, numerous clinical trials using a variety of MDR-1 inhibitors failed to show significant clinical benefits, despite the evidence supporting a role for MDR-1 in drug resistance, (reviewed in [11,77,78,79]). This could be due to ABC receptor redundancy as there are many drug transporters encoded within the human genome (members of the ABCB, ABCC, and ABCG subfamilies), and expression of just one specific efflux pump could be enough to facilitate drug resistance.

Our observations are consistent with the idea that drug efflux pumps can be redundant, having found that drug resistant cells lines derived from a variety of tumor types express either MDR-1 or BCRP *in vitro*, but not necessarily both [22]. Our results also support the hypothesis that MDR-1 may at the very least be a marker of aggressive tumors [78], as we consistently observe at least MDR-1 or BCRP protein expression in all tumors samples obtained from human breast cancer patients and canines with lymphoma that failed chemotherapy [80]. 

Interestingly, a second mechanism whereby ABC transporters contribute to treatment resistance is emerging independent of their inherent drug efflux function, and may in fact also reflect the high energy costs of “running” these ABC transporter pumps. For example, the amount of ATP hydrolyzed for each drug transported is far more than what is expected; *in vitro* biochemical studies suggest that approximately two molecules of ATP are hydrolyzed per drug transported [81,82], yet reconstituted systems show that MDR-1 continues to hydrolyze ATP even in the absence of drug [83]. This clear inefficiency of the ABC system raises the question of how cancer cells adapt metabolically to fuel these abundant transmembrane pumps (reviewed in [70,84]).

### 4.2. Metabolic Shifts towards Glycolysis and Proton Pumping in Cancer Cells Promote MDR

Cancer cells undergo defined metabolic shifts that distinguish them from normal cells, including adaptation from oxidative (oxygen and mitochondria dependent metabolism, maximize the ATP per glucose produced) to hypoxic (independent of mitochondria and their pH gradients) microenvironments. There is an initial shift from glycolysis to respiration regardless of available oxygen and glucose, known as the Warburg effect [85,86,87], followed by acidification of the extracellular microtumoral environment with corresponding intracellular alkalinisation, referred to as pH gradient reversal [70,88,89]. When pH gradient reversal occurs, cytosolic vesicles, such as lysosomes, are maintained at a very low pH within the higher pH intracellular milieu [90,91,92]. Interestingly, studies in model organisms and in cancer cells demonstrate that the longer the lysosome/vacuole remains active (pH remains low), the longer the organism or tumor cell will survive [93,94,95]. This leads to the notion that enhanced vacuolar acidic pH may be a major contributing factor allowing aggressive tumors to thrive. The alkaline pH within the cytosol facilitates glycolysis, with current thinking suggesting that it is the malignant rise in intracellular pH that is behind the Warburg effect (reviewed in [89]). Whether by design or not, the switch to glycolysis allows a cell within a solid tumor to adapt to the heterogeneous hypoxic and low nutrient conditions present within many tumor microenvironments. An acidic and hypoxic environment deregulates normal apoptotic control through the activation of several pH sensitive cellular transporters and kinases that, in contrast, mediate proliferation, angiogenesis, and invasion.

It has also been hypothesized that the reversal of the pH gradient across the plasma membrane in cancer cells may also play an important role in MDR development and maintenance [96,97,98]. In fact, inhibition of the ABCB1 drug efflux pump MDR-1 using verapamil lowered intracellular pH in drug resistant lung tumor cells [96,97,98], suggests that MDR-1 may play a direct role in the reversed pH gradient promoting the drug resistant state. It has also been suggested that the acidic extracellular pH of the tumoral microenvironment may itself block the passage of drugs [97,99]. For example, drugs that are weak bases, such as DOX, are neutralized and inactivated by the acidic environment in which the tumor cells are imbedded. Hypoxia and low pH environments also lead to the expression of HIF1α, which in turn promotes angiogenesis, proliferation, and metastasis by increasing the expression of vascular endothelial growth factor (VEGF) [100]. Additional work also demonstrates that HIF1α is critical for the switch from oxidative phosphorylation to fermentative glycolysis (reviewed in [101,102,103]). Considering our discussion above regarding MDR development, together with the role of HIF1α in MDR maintenance, a model becomes clear. We showed that the anticoagulent protein TFPI1 becomes overexpressed upon exposure to high DOX concentrations, generating a hypoxic-like state by abrogating the expression of an angiogenic profile, and thus inducing the expression of a host of cancer driving proteins including HIF1α [22]. We propose that the induction of a hypoxic-like state by TFPI1 is sufficient to possibly initiate a pH gradient reversal and a switch to glycolysis, as well as induction of a secondary angiogenic pathway mediating by HIF1α. Based on this model, it seems at least promising that combining compounds that inhibit HIF1α with first-line therapy, such as DOX, should have beneficial effects.

## 5. Reversal of MDR

### 5.1. Drug Efflux Pump Inhibitors

Referring to our concept of three distinct phases of MDR, MDR reversal implies the resensitization of treatment resistant cells to therapy (*i.e.*, DOX). Proteins identified as being critical for MDR development and/or maintenance, if deemed druggable, will provide a rational choice for drug discovery and design. An enormous amount of work has been dedicated to identifying druggable targets that will halt the proliferation of MDR cells (reviewed in [104,105]). Reasonably, much of the work has focused on developing inhibitors against the drug efflux pumps MDR-1 and BCRP, which show promise against breast and other cancers *in vitro* [106,107,108]. Studies using mouse models where ABC transporters are inhibited are also beginning to show promise as a reversal strategy [109,110,111]. However, inhibitors against ABC transporters have not met with success in the clinic for a variety of reasons, such as silent polymorphisms present in the population [79,112,113,114,115].

### 5.2. HIF1α Inhibitors and Hypoxia

Strategies against other targets have also been used in an attempt to reverse the MDR phenotype, including the development of inhibitors against HIF1α. HIF1α expression is tightly correlated with transformation into aggressive tumors, as discussed above [65,66,67]. HIF1α protein is rapidly expressed and stabilized under hypoxic conditions, as well as in the presence of other stresses such as nitric oxide, cytokines, insulin growth factors, and expression of oncogenes [116]. It has been predicted that drugs that block HIF1α expression, stability and/or activity, should synergize with first-line chemotherapy due to their different mechanisms of action (reviewed in [21]). Blocking the PI3K/Akt/mTOR pathway (which indirectly activates HIF1α), with the inhibitor, temsirolimus has shown promise *in vitro* and in phase II clinical trials against breast cancer [115,117]. Additional support for targeting HIF1α to combat cancer is based on observations that anti-angiogenic therapies, while initially beneficial, will ultimately induce the expression of HIF1α [118]. Blocking the expression of HIF1α should, in theory, block its induction of a cascade of proteins associated with cancer invasion, proliferation, and metastasis. We have also observed an induction of HIF1α in response to therapy, as noted *in vitro* when MCF7 cells are treated with high doses of DOX [22]. Recent preclinical, phase I, and phase II clinical trials have shown promising results when HIF1α inhibitors are combined with chemotherapeutic agents [119,120,121]. Further studies are required to fully evaluate the effects of HIF1α inhibitors in phase III clinical trials and may eventually prove to be effective therapeutic options.

### 5.3. Poly (ADP-Ribose) Polymerase Inhibitors and Hypoxia

Inhibitors against poly (ADP-ribose) polymerase (PARP) have also generated interest over the past decade [122]. PARP is a molecular sensor of DNA breaks and acts to promote cell survival by facilitating DNA break repair. It functions in the surveillance and maintenance of genome integrity by adding poly ADP-ribose to partner proteins, such as histones, bound to the broken DNA ends. Polyribosylation of histones contributes to the relaxation of the 30 nm chromatin fibre, thus increasing access to the free DNA ends for repair proteins, and it acts as a counter of damaged DNA in order to mount an adaptive and appropriate response.

Inhibition of PARP was found to act in a synthetically lethal manner with BRCA1 and BRCA2 mutations, which themselves block homologous DNA recombination-repair *in vitro*, with clinical benefits observed in clinical trials [123,124]. *In vitro* cell biology has revealed that hypoxia inhibits both the homologous DNA repair pathway and BRCA1 translation, supporting the idea that PARP inhibition may be effective in treating tumors engulfed in a hypoxic environment, such as solid breast tumors [21,125].

### 5.4. Histone Deacetylase Inhibitors and Hypoxia

Another highly effective class of inhibitory compounds tested in a variety of clinical trials are the histone deacetylase inhibitors (HDACi’s; reviewed in [126,127]). Use of these compounds results in a general increase in global histone acetylation, which subsequently results in increased global transcription and an antiproliferative effect. The mechanism contributing to HDACi’s antiproliferative effects have been studied by many, and likely stems from their ability to induce the expression of genes involved in cell cycle arrest, DNA repair, free radical scavenging, while blocking the phosphorylation and activity of the procancer cancer PI3K/Akt/mTOR pathway [128,129]. Of relevance to this review, HDACi’s have been shown to block hypoxia-stimulated signaling and HIF1α function by inhibiting HIF1α transcriptional activity and stability [130,131,132]. This has been shown to rely on increased expression of HDACs in the presence of hypoxia [101,133]. Indeed, HDAC2 is considered a tumor promoting protein, with inhibition resulting in cell death, effectively reducing tumor growth [134].

### 5.5. Anti-Diabetic Drugs Have Potential against MDR Cancers

The thiazolidinedione (TZD) family of compounds are prescribed for those with Type 2 Diabetes mellitus (DM2) [135]. The TZDs are most often considered as peroxisome proliferator-activated receptor gamma (PPAR-γ) ligands that activate PPAR-γ. Troglitazone (TRG) was used clinically until it was pulled from the market in 1998 due to the potential of severe liver toxicity [136,137]. Rosiglitazone (ROSI) and Pioglitazone (PIO) use was widespread until 2010, when ROSI specifically was pulled from the market due to an increased risk of cardiovascular events. PIO remains as the only TZD in current use [138]. Of this TZD class, it was discovered in 1998 that TRG had potent antiproliferative potential when used *in vitro* to treat MCF7 breast cancer cell lines [139].

We extended these observations by showing that TRG synergized with the anthracycline DOX to inhibit the growth of K562 human leukemia cells and MCF7 breast cancer cells previously selected for DOX resistance [23,24], implicating TRG in the resensitization of resistant cells to therapy. TRG exposure caused global histone acetylation to increase in K562 and MCF7 cells, which we showed was due to direct HDACi activity [24,140]. TRG was the most effective antiproliferative of the TZDs tested (which included PIO and ROSI) and was the only drug to exert HDACi activity [140]. This may be due to TRG’s unique structure that differs from ROS and PIO. During the development of TRG in the early 1990s, an intact α-tocopherol (Vitamin E) moiety was incorporated to avoid lipid peroxidation in the liver [141]. Analogues of Vitamin E have the capacity to induce apoptosis and synergize with other anticancer agents to inhibit the growth of breast cancer cells *in vitro* [142,143]. Furthermore, Vitamin E metabolites possess structural qualities that suggest HDACi activity [144]. Moreover, TRG’s HDACi activity may be derived from quinone derivatives that are TRG metabolites [145,146]. Quinone derivatives are thiol-reactive, and thiol-reactive compounds, such as isothiocyanates, have demonstrated HDACi activity [147].

### 5.6. Resensitizing Treatment Resistant Cancer by Multiple Mechanisms

Also of note was our observation that TRG exposure in DOX resistant MCF7 and K562 cells significantly decreased the protein levels of the ABC transport efflux pumps MDR-1 and BCRP over a relatively short time period [23]. This is relevant to TRG-dependent killing in MCF7 DOX resistant cells, as partial silencing of MDR-1 increased the sensitivity of these cells to a subsequent exposure to DOX [23]. This work showed that members of the TZD class of anti-diabetic drugs (TRG) have the potential to inhibit at least two cellular mechanisms that respond to hypoxia: HDACs and drug efflux pumps. TRG therefore potentially acts to reduce the defenses of the drug resistant cells, akin to “lowering the shields” of these aggressive cells.

### 5.7. TRG Reverses the Down-Regulation of a 40-Gene Cluster in DOX Selected Cells

**Figure 2:**
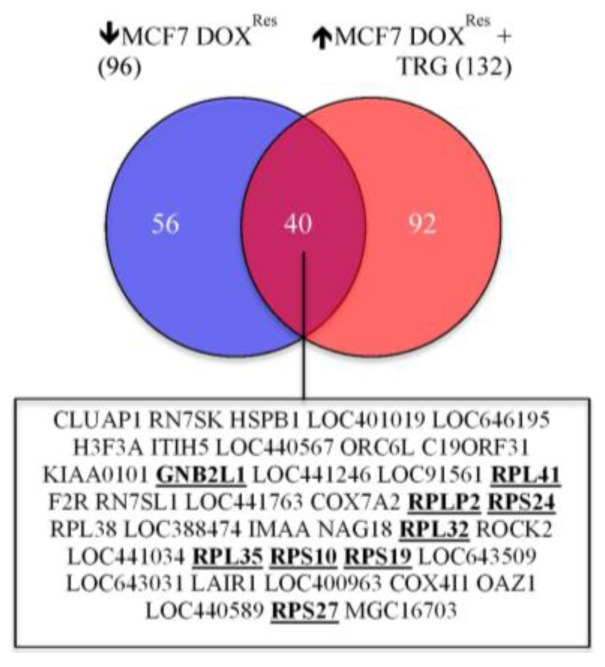
Identity of 40 genes down-regulated during selection for Doxorubicin resistance in MCF7 cells (MCF7 DOX^Res^) that are up-regulated upon treatment with Troglitazone (TRG). The bolded/underlined genes represent a cluster involved in ribosome assembly [80].

**Figure 3:**
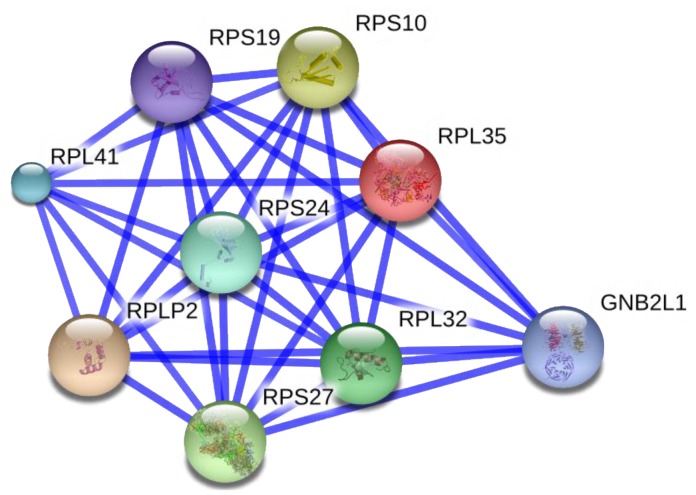
TRG up-regulates the expression of 40 genes that were down-regulated during the development of resistance to DOX. A cluster of nine of these genes (eight ribosome subunits and one regulatory factor, GNB2L1) interact within a network involved in ribosome biogenesis, as determined using the STRING database (version 9.1; http://string-db.org). The Confidence View is shown with the width of the connecting lines indicating increased confidence of the interaction [80].

As an extension of our microarray study identifying differentially expressed genes specific to drug selection [22], we performed a microarray analysis on the DOX selected cells after subsequent treatment with TRG (unpublished). A unique subset of 40 genes were downregulated upon development of DOX resistance, and subsequently amplified upon TRG treatment in the same cell population (Figure 2), suggesting that these genes are critical to the operative mechanism(s) for MDR development. These forty genes were enriched for a cluster of 9 genes involvement in ribosomal biogenesis (eight ribosome subunits and one ribosome regulator, GNB2L1/RACK1: Figure 3). Thus, a decrease in the protein products of genes involved in ribosome biogenesis would be predicted to correlate with the development of MDR, and their reappearance would coincide with reversal of resistance (resensitization).

### 5.8. Role of Hypoxia and TFPI1 in the Down-Regulation of Ribosome Biogenesis in the Development of DOX Resistance

Ribosomal biogenesis-related genes (9 of the 40 TRG up-regulated genes described above) were prominently represented within this list (bolded in Figure 2). Although our hypothesis that down-regulation of ribosomal biogenesis is important for MDR development is currently based on our unpublished data, it is supported by observations described in the literature. It has been suggested that alterations in ribosome biogenesis may play a critical role in stress response and progression of cancer [148,149]. For example, development of cancer is often associated with increased ribosome biogenesis in order to maintain the high metabolic load of these cells. Large irregular shaped nucleoli have long been associated with cancer progression, with first observations occurring in the late 1800’s (reviewed in [150]). If so, how does reduced expression of ribosome biogenesis genes play a role in the development of resistance to DOX as suggested by our findings? Reduced expression of ribosome proteins has indeed been reported to cause tumors, which has been examined at length in patients with inherited genetic diseases called ribosomopathies [151,152]. Nonetheless, it remains unclear why individuals afflicted with ribosomopathies would be at a greater risk for cancer. Decreased protein translation could certainly induce a stress response, which may include the induction of HIF1α. HIF1α is a stress response protein known to respond to many stresses other than just hypoxia [116]. If this were to occur, then it becomes possible to imagine how reduced protein translation could lead to increased cancer risk.

An additional link with reduced ribosome function and drug resistance could be hypothesized to involve the onset of hypoxia. Protein translation in normal cells is inhibited by hypoxia [153]. Hypoxia activates the AMP-dependent protein kinase (AMPK), which blocks CAP-dependent translation of mRNAs by inhibiting TOR-dependent translation. The elevated protein translation observed in tumor cells is driven by constitutively expressed c-MYC [154]. When tumor cells are exposed to hypoxia, a new equilibrium is sought between HIF1α and c-MYC for control over translation. As HIF1α expression increases, protein translation is reduced, even in the presence of c-MYC [154].

Another effect of hypoxia on protein translation is the EIF4G1-dependent switch from CAP-dependent translation to CAP-independent translation, resulting in the selective translation of mRNAs containing internal ribosomal entry sites found in mRNAs encoding proteins such as VEGF, HIF1α, and the fibroblast growth factor 2 [155]. However, it is felt that CAP-independent translation cannot fully account for the translational capacity of hypoxic cells, as recent studies show that HIF1α and HIF2α can drive the CAP-dependent translation of a subset of mRNAs required for breast cancer cell growth [156,157]. Nonetheless, we found that the CAP-independent translation initiation factors EIF3I and EIF4G1 were elevated in DOX selected cells [22], and are found overexpressed in many advanced breast tumors [155]. Thus, our microarray data supports the idea that the hypoxic-like microenvironment initiated by DOX, and potentially induced by TFPI1 and the anticoagulant pathway, may in part facilitate MDR development by reducing ribosome assembly gene expression.

Further evidence to suggest that TFPI1 may play a role in altering ribosome biogenesis was our observation that TFPI1 relocates from its resting state plasma membrane position [59] to the nucleolus in MCF7 DOX^Res^ cells [22]. Whether TFPI1 nucleolar enrichment is linked to MDR development, or oncoprotein induction, is unknown, but nucleoli are home to ribosomal assembly and we have identified the down-regulation of many ribosomal biogenesis genes in our MDR development screen, suggesting that such linkages may be integral to these processes. Such alterations to ribosomal pathways may introduce pressure on the nucleolar machinery, triggering the Nuclear Stress Pathway that can be experimentally detected as decreases in ribosomal biogenesis and activation of p53 [158,159]. Our observation that TFPI1 translocates from the plasma membrane and cytosol to the nucleolus when cells are treated with high dose DOX is novel, and will require further study to fully understand.

### 5.9. Metformin as an MDR Sensitizer

Another class of antidiabetic drug, the biguanides, of which Metformin (MET) is a member, has been shown over the past decade to have antiproliferative potential against cancer cells, *in vitro* and *in vivo* [160,161]. Indeed, many retrospective meta-analyses have shown that MET possesses anti-cancer activities and decreases the incidence of primary cancer development in those taking MET routinely [162,163,164,165,166], and a multitude of clinical cancer trials are actively assessing its benefits in the non-diabetic population who have already developed cancer [167,168]. The mechanisms of action for MET in metabolic control (*i.e.*, DM2) are well-studied; MET is known as an insulin sensitizer with multiple effects on metabolism, primarily through AMPK activation via inhibition of mitochondrial Complex I [169,170,171,172]. MET is thought to directly or indirectly mediate the phosphorylation of LKB1 targets, such as AMPK, under times of stress, including hypoxia. MET may also be effective against hypoxic embedded solid tumors by reducing O_2_ consumption within these cells [173].

However, the precise molecular mechanisms whereby MET works in cancer prevention remain multifactorial and ill-defined. Downstream effects of MET action can mimic caloric restriction and include inhibition of the PI3K/Akt/mTOR pathway and induction of DNA repair mechanisms, which may rely on AMPK activation [169,174,175]. Future studies are required to elucidate the role of MET as adjuvant therapy in newly diagnosed cancer and in cancers exhibiting drug resistance.

## 6. Summary and Future Directions

This review has summarized the current literature that is exploring the mechanisms of multiple drug resistance development, maintenance and reversal and emerging treatment strategies to overcome MDR. Given the potential clinical benefits to be gained from blocking these hypoxia-stimulated pathways, a variety of inhibitors have been developed against multiple mechanisms to block the effects of hypoxia triggered by exposure to chemotherapeutic compounds. Clinical trial-assessment of many of these inhibitors are either awaiting to be done or in the early stages and therefore has yet to translate to clinical benefits, as the population of individuals with MDR cancer of the breast or otherwise, remains without effective, non-toxic therapeutic options. Further development of new or repurposed compounds against treatment resistant cancers embedded in hypoxic and acidic milieus is paramount.

Also important to address is the still unfulfilled need to be able to detect the development or inherent presence of the MDR phenotype in cancer patients before there is clinical failure. Future work in MDR cancer biology will require the critical unveiling of early, valid biomarkers of MDR to appreciate that first line therapeutic management strategies may be futile and to avoid ineffective toxic treatments. Even more significantly, understanding the cancer biology of MDR development and establishment will ultimately elucidate novel molecular targets capable of preventing MDR development in the first place, or in reversing existing/inherent MDR so that patient outcomes can be dramatically improved with first line therapeutic options with tolerable toxicities.
